# Safety and Efficacy of Topical Lotilaner Ophthalmic Solution 0.25% for the Treatment of *Demodex* Blepharitis: A Pilot Study

**DOI:** 10.1155/2021/3862684

**Published:** 2021-09-21

**Authors:** Roberto Gonzalez-Salinas, Elizabeth Yeu, Mark Holdbrook, Stephanie N. Baba, Juan Carlos Ceballos, Martha Massaro-Corredor, Claudia Corredor-Ortega, Nallely Ramos-Betancourt, Hugo Quiroz-Mercado

**Affiliations:** ^1^Asociación Para Evitar la Ceguera en México I.A.P., Mexico City, Mexico; ^2^Virginia Eye Consultants, Norfolk, VA, USA; ^3^Tarsus Pharmaceuticals, Inc., Irvine, CA, USA

## Abstract

**Purpose:**

Evaluate safety and efficacy of topical lotilaner ophthalmic solution, 0.25% for the treatment of *Demodex* blepharitis. *Patients and Methods*. 15 patients with *Demodex* blepharitis, defined as >10 collarettes on the upper lid, lid margin erythema, and *Demodex* density of ≥1.5 mites/lash on microscopy, were treated *bid* for 28 days with lotilaner ophthalmic solution, 0.25%. Contact lens wear, artificial eyelashes, and lid structural abnormalities were among the exclusion criteria. No other antibacterial, antiparasitic, or anti-inflammatory treatment or lid hygiene products were permitted. Patients were assessed on Days 7, 14, 28, 60, and 90. Outcome measures were changes in collarette grade and mite density on Day 28. Adverse events and changes in intraocular pressure (IOP), corrected distance visual acuity (CDVA), and slit-lamp biomicroscopy were assessed.

**Results:**

Mean collarette grade (upper lids) improved from 3.07 ± 0.21 to 0.79 ± 0.19 on Day 28; the change was statistically significant for both upper and lower lids from Day 14 on. Mean mite density per lash decreased from 2.28 ± 0.16 at baseline to 0.14 ± 0.05 at Day 28 (*p* < 0.0001). Mite eradication (0 mites) was documented in 57.1% of eyes. The effects were durable through Day 90. There were no adverse events and little to no change in CDVA or IOP. The drop was well tolerated, with no discontinuations due to ocular irritation.

**Conclusion:**

Topical lotilaner ophthalmic solution, 0.25% for 4 weeks, showed promising efficacy for the treatment of *Demodex* blepharitis. This novel treatment appears to be safe and well tolerated. Randomized controlled studies are needed to confirm the results.

## 1. Introduction

Blepharitis is a chronic, progressive condition characterized by inflammation, ocular irritation, and erythema [1]. If left untreated, it can lead to more significant sequelae, including lid and lash abnormalities, blurred vision, and corneal damage. Blepharitis primarily affects the lid margin but may also affect the eyelid skin, base of the eyelashes, eyelash follicles, and the meibomian glands and gland orifices. Up to 20 million US adults may have blepharitis.

The association between blepharitis and *Demodex* infestation has been examined in several studies. In a meta-analysis of 13 controlled studies, Zhao et al. reported that the rate of *Demodex* infestation in blepharitis patients is 44.5%, compared to 16.7% in normal controls [2]. Biernat et al. also reported that the prevalence of ocular *Demodex* infestation is significantly correlated with blepharitis [3].

*Demodex* mites are the most common ectoparasites found on human skin and eyes. There are two species: *Demodex folliculorum* burrows into hair, thus eyelash and follicles, while *Demodex brevis* prefers to inhabit the sebaceous and meibomian glands [4, 5]. Although *Demodex* mites are commonly part of the mammal ocular flora, an overpopulation or infestation of mites (ocular demodicosis) occurs only in humans and is associated with ocular surface inflammatory conditions [6]. *Demodex* infestation increases with age [3, 7–9] with nearly all of those over age 70 experiencing the condition [8].

*Demodex* mites contribute to blepharitis inflammation through mechanical damage as they burrow and lay eggs; chemical irritation from their digestive enzymes and the waste products released upon mite death; and bacterial contamination [5]. As the mites feed on eyelid skin and hair follicle cells and proceed through their life cycle, the partially digested epithelial cells, waste, and eggs form collarettes (cylindrical dandruff) at the base of the lashes. These collarettes are now recognized as pathognomonic for *Demodex* blepharitis [9, 10].

Currently, there are no FDA-approved treatments for blepharitis due to *Demodex* infestation. The American Academy of Ophthalmology Preferred Practice Pattern (PPP) for blepharitis suggests a combination of antibiotics, topical anti-inflammatory agents, and daily lid hygiene [1]. The most common lid hygiene approach involves the use of scrubs, wipes, or gels containing tea tree oil (TTO) or its major acaricidal component, terpinen-4-ol (T4O). TTO and T4O products, with a wide range of concentrations, have been tested in a number of studies [11–16]. A recent Cochrane review found that their efficacy was uncertain [16]. In addition, TTO can be toxic to epithelial cells and fibroblasts [17, 18] and has recently been shown to be harmful to human meibomian gland epithelial cells *in vitro* [19].

Lotilaner belongs to the isoxazoline class of parasiticides with origins in veterinary medicine. When exposed to isoxazolines, ectoparasites exhibit spastic paralysis leading to their starvation and death [20]. Both the U.S. Food and Drug Administration (FDA) [21] and the European Medicines Agency (EMA) [22] have concluded that isoxazoline veterinary products are safe and effective. A 2019 FDA advisory noted that the isoxazoline parasiticide class has been associated with neurologic adverse reactions. These adverse reactions are very rare and no specific hypothesis for the responses in the affected animals has been proposed [21, 22]. Specific to lotilaner, there have been no reports of neurotoxicity in the completed nonclinical toxicology studies and there were no clinical reports of neurotoxicity in the studies supporting approval of lotilaner for veterinary use, nor in any human studies to date. Lotilaner exhibits highly selective antagonist activity against insect GABA-Cl channels [23] but has negligible activity on mammalian GABA-Cl channels. Specifically, lotilaner has been reported to exhibit greater than 10^4^-fold higher affinity to invertebrate GABA-Cls and Glu-Cls compared to mammalian channels [24]. *In vitro* functional characterization of lotilaner against several dog [24] and human (unpublished research) GABA-Cl receptors confirmed that lotilaner had no significant inhibition at concentrations up to 30 *μ*M. As such, lotilaner is the isoxazoline parasiticide that has the least potential to have any neurologic adverse effect on mammals among all commercially available veterinary isoxazoline parasiticides.

Lotilaner has not previously been studied in humans. Preclinical, *ex vivo* testing demonstrated that lotilaner 0.25% killed >95% of *Demodex* mites within 24 hours [25].

This pilot study, the first clinical study in humans, was conducted to evaluate the safety and efficacy of a topical ophthalmic formulation of lotilaner for the treatment of blepharitis due to *Demodex* infestation.

## 2. Materials and Methods

This was a single-arm, open-label, Phase 2a treatment study. The study was approved by the Asociación para Evitar la Ceguera en México I.A.P. Committee on Ethics in Investigation and conducted in accordance with the ethical principles of the Declaration of Helsinki. Fifteen patients with blepharitis due to *Demodex* infestation were enrolled, treated twice daily for 28 days, and followed up for a total of 90 days.

Male and female adult patients (≥18 years old) who were willing to sign the informed consent and deemed capable of complying with the study protocol requirements were eligible to be enrolled. For inclusion in the study, patients had to have >10 collarettes present on the upper lid; mild to severe lid margin erythema, and *Demodex* mite density of ≥1.5 mites per lash on microscopy in at least one eye.

Contact lens wear, artificial eyelashes, and eyelash extensions were not allowed during the study treatment period or for 7 days before the baseline screening visit. No systemic or topical antibacterial, antiparasitic, or anti-inflammatory treatments; topical tea tree oil or hypochlorous acid; or other lid hygiene products were permitted during the treatment period or for 14 days before the screening visit. Patients were excluded if they had used a topical prostaglandin analogue (PGA) to promote eyelash growth, had initiated PGA treatment for medical reasons within the past 30 days, or planned to change or discontinue prostaglandin treatment for medical reasons during the study.

Other exclusion criteria included previous surgery of the lid margin; lid structural abnormalities that could influence the study; acute ocular infection; active inflammation other than blepharitis; severe dry eye; pregnancy; and unstable or uncontrolled systemic disease. Patients were excluded if they had any known sensitivity or allergy to lotilaner or any of the formulation ingredients.

Patients were treated with an eye drop formulation containing the active ingredient lotilaner 0.25% (TP-03, Tarsus Pharmaceuticals, Irvine, CA, USA) and other inactive ingredients in a preserved, multidose drop bottle. The first two doses were administered in the clinic, after which the drops were dispensed to the patient for use at home. They were instructed to instill one drop in each eye, twice daily.

At the screening visit, potential participants were evaluated for eligibility, including confirmation of *Demodex* infestation. Collarettes and lid margin redness were assessed, mites were counted under the microscope, and slit-lamp photographs of the upper and lower lid margins were obtained. A pregnancy test was administered to females of child-bearing age. Written informed consent was obtained prior to the administration of any study medication or testing. For those qualified to participate, the first dose and additional Day 1 testing could take place on the same day as the screening visit or at another visit within 14 days.

After an initial dose on Day 1, patients returned the following day for a complete safety evaluation and administered the second dose under supervision in the clinic on Day 2. The remainder of the drops were instilled by the patients at home.

Patients were assessed on Days 7, 14, 28, 60, and 90. At each visit, corrected distance visual acuity (CDVA) and intraocular pressure (IOP) were measured and slit-lamp biomicroscopy, including assessment of collarettes, was performed. Collarettes were evaluated and assigned a grade from 0 to 4 (0 = no collarettes, 1 = 1 − 10 collarettes per eyelid; 2 = >10 but less than 1/3 of the lashes with collarettes; 3 = ≥1/3 but <2/3 of lashes; and 4 = ≥2/3 of the lashes). Magnified images focused on the lashes and lid margins were obtained at each visit.

*Demodex* mites were counted under the microscope at baseline, Day 14, and every visit thereafter. Two or more lashes each were removed from the upper and lower eyelids (selecting those with collarettes if present). Lashes were placed on glass slides, covered with an artificial tear with an emulsifier, and placed under a coverslip under the microscope so the mites could be seen and counted.

One eye of each patient was selected as the analysis eye. The analysis eye was the eye that met all inclusion criteria. If both eyes met all inclusion criteria, then the analysis eye was the eye with the highest *Demodex* density at the screening visit or, if both eyes had equal *Demodex* density, the right eye.

Efficacy outcome measures included the improvement in collarette grade at Day 14 and Day 28 and the change in *Demodex* density from baseline to Day 28. Safety was determined by assessing adverse effects related to the treatment, as well as evaluating any changes in CDVA, IOP, or slit-lamp biomicroscopy findings.

Safety measures were summarized using descriptive statistics. Efficacy measures were analyzed using paired analyses for the change from baseline, paired *t*-test, or Wilcoxon signed-rank test as appropriate. Comparisons were one-sided using an *α* of 0.05. No adjustment was made for multiple comparisons.

## 3. Results

Eighteen patients were enrolled. Three patients received a single dose of the study medication but failed to return on Day 2 and were not dispensed any drops for use at home. These patients were not included in the study. Of the remaining 15 patients, 1 was lost to follow-up after Day 2; 1 patient missed the Day 7 visit, but continued with the remaining follow-up visits; and 1 patient missed Days 14 and 28 visits. This patient's Day 28 results, other than safety results, were imputed from the Day 60 visit.

Patients ranged in age from 44 to 89 years (mean 69.5 ± 13.2). Demographic and baseline characteristics are shown in Table 1.

**Figure 1:**
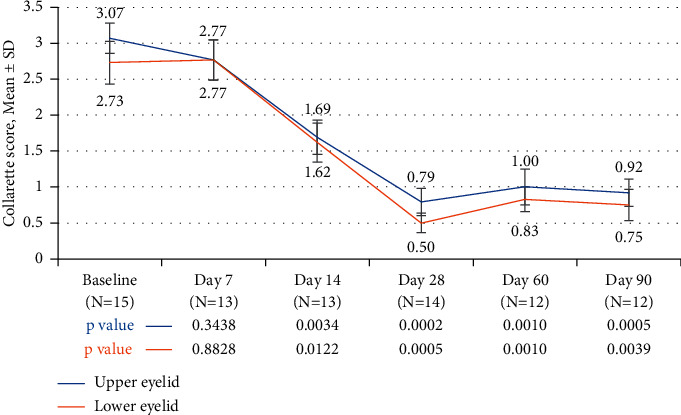
The difference in collarette grade from baseline was statistically significant for both upper and lower lids of analysis eyes on day 14 and every visit thereafter.

**Figure 2:**
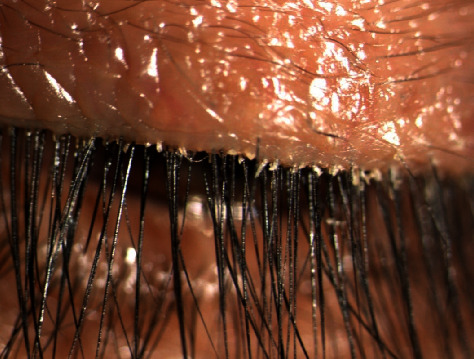
A magnified image of the upper lid margin in a representative eye (left eye of patient 009) shows a corrugated lid margin with significant collarettes.

**Figure 3:**
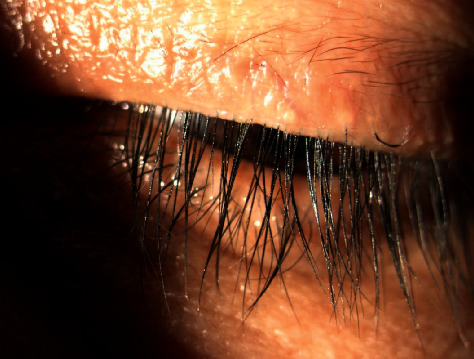
Following treatment with lotilaner ophthalmic solution, 0.25% for 28 days, the lid margin in the left eye of patient 009 is greatly improved, with no evidence of collarettes.

Collarette grade was calculated separately for the upper and lower lids of the analysis eyes. The mean collarette grade for the upper lids improved from 3.07 ± 0.21 at baseline to 1.69 ± 0.24 at Day 14 and 0.79 ± 0.19 at Day 28. The difference in collarette grade from baseline was statistically significant for both upper (*p*=0.0034) and lower (*p*=0.0122) lids at Day 14 and every visit thereafter (Figure 1). Figures 2 and 3 show a representative example of eyelash collarettes before and after treatment.

**Figure 4:**
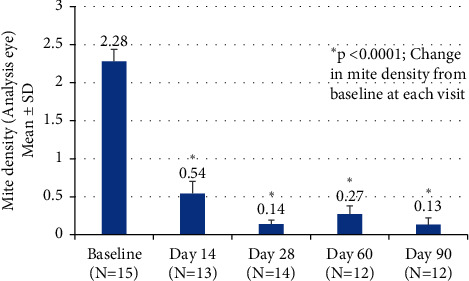
Mean mite density per lash decreased from 2.28 ± 0.16 at baseline to 0.14 ± 0.05 on Day 28 (*p* < 0.0001) in the analysis eyes. The improvement was maintained through Day 90.

**Figure 5:**
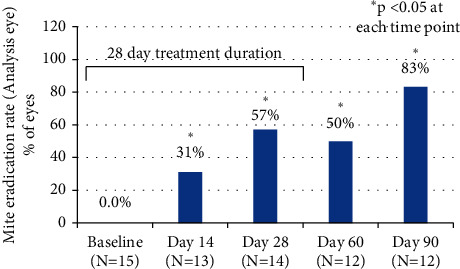
Complete eradication of mites (0 mites) was observed in 8 of 14 eyes (57.1%) on Day 28 and 10 of 12 eyes (83.3%) on Day 90.

Mean mite density per lash decreased from 2.28 ± 0.16 at baseline to 0.14 ± 0.05 at Day 28 (*p* < 0.0001). The improvement in mite density was maintained through Day 90, approximately 2 months after discontinuation of *bid* treatment (Figure 4). The change in mite density from baseline was statistically significant at all visits. Complete eradication of mites (0 mites) was observed in 8 of 14 eyes (57.1%) by Day 28 (Figure 5).

There were no adverse events. There was little to no change in mean CDVA during the study. Five patients experienced clinically significant (0.2 logMAR) improvement in CDVA at one or more visits. No patient lost 0.2 logMAR CDVA. There were no significant changes in IOP during the study.

Five patients had mild increases in corneal staining, which improved at subsequent visits. One patient had mild, transient, bilateral conjunctival hyperemia. No patients discontinued treatment due to any medication intolerance.

## 4. Discussion

This pilot study is the first clinical evaluation of topical lotilaner ophthalmic solution, 0.25% in humans. The study drug was formulated at its maximum dosage for an aqueous solution. Based on preclinical studies performed for regulatory approval as a veterinary medication, the no-observed-adverse-effect level (NOAEL) for lotilaner in a 13-week oral administration study in rats was determined to be 20 mg/kg/day. Assuming a 35 *μ*L drop size, four drops a day of 0.25% lotilaner (2 drops per eye) would provide a dose of 0.35 mg/day. For a 60 kg patient, that would be a dose of 0.006 mg/kg/day or less than 0.03% of the preclinical NOAEL [26]. In preclinical work in rabbits, when TP-03 was dosed in concentrations up to 0.25% TID for 28 days, the concentration and dosage regimen were well tolerated.

Topical treatment with lotilaner ophthalmic solution, 0.25%, resulted in statistically significant decreases in the collarette grade, for both upper and lower eyelid margins, at Days 14 and 28 (Figure 1). The decrease persisted for the duration of the study and at least 2 months following treatment. Collarettes are an important outcome measure. Not only have recent studies shown a strong correlation between collarettes and *Demodex* infestation [9] but collarettes also can be easily identified during a standard eye examination.

Another outcome measure was the change in *Demodex* mite count by microscopy during the 28-day treatment period. We found statistically significant decreases in mite density at Days 14 and 28, with the decreases maintained throughout the duration of the study (*p* < 0.0001 at all time points). Complete mite eradication was seen on microscopy in 57.1% of the analysis eyes at Day 28. Few studies of other treatments for *Demodex* blepharitis have reported mite reduction to zero. Fromstein et al. noted that even among lid hygiene products intended to address signs and symptoms of *Demodex* blepharitis, no product has been shown to fully eradicate mites at 4 weeks [5]. Koo et al. reported an overall *Demodex* mite eradication rate of 23.6% at one month in patients treated with weekly in-office lid scrubs with high-concentration TTO in addition to twice-daily, at-home lid scrubs [27]. This treatment regimen was quite arduous compared to the self-administered topical drop used in our study. It should also be noted that TTO lid hygiene is known to cause ocular irritation [27–30] and contact dermatitis [31].

Two recent reports have investigated the use of intense pulsed light (IPL) in a series of three to four treatments on patients with *Demodex* blepharitis. [32, 33]. However, the reduction of mite density with IPL seems to be variable. Zhang et al. compared IPL with 5% TTO and did not find significant effects in terms of mite counts in their study but did note differences in OSDI, TBUT, and meibum quality [33]. In another study, Cheng et al. reported only a 20% mite eradication rate with IPL [32]. IPL cannot be performed on patients with darker skin tones (Fitzpatrick V or VI).

Patients in the current study reported very good tolerability of the lotilaner eye drops, with no adverse events or other safety concerns, at least in this small sample. In future controlled studies in a larger number of eyes, it will be important to continue to evaluate tolerability.

We know that *Demodex* infestation has been implicated in ocular surface inflammation [6] and that treatment of *Demodex* infestation has been shown to reduce the concentration of inflammatory cytokines and chemokines in the tear film [34]. The impact of lotilaner therapy on objective measures of the lid or conjunctival redness and lid or tear film inflammation may be of interest for future study.

The ideal duration of treatment for *Demodex* blepharitis is not known. Patients in this pilot study were treated for 28 days and followed up for an additional 2 months after treatment (90 days total). We began to see statistically significant efficacy as early as 14 days. Efficacy, as measured by both collarette score and mite density, was maintained through the 90-day follow-up period. A treatment duration longer than 28 days may be beneficial. Additionally, a longer-term follow-up of 6–12 months in future studies would be helpful to know if and when collarettes and mites recur.

## 5. Conclusions

There is currently no efficient, effective topical treatment available for *Demodex* blepharitis. In this study, topical treatment with lotilaner ophthalmic solution, 0.25% for 4 weeks, showed promising efficacy for the treatment of blepharitis due to *Demodex* infestation. The beneficial results observed during the treatment period persisted for at least 2 months following treatment. This novel treatment appears to be safe and well tolerated. Randomized, controlled studies in larger populations are needed to confirm the results.

## Figures and Tables

**Table 1:** Demographics and baseline characteristics.

Age	
Mean (SEM)	69.5 (13.2)
Sex	
Male, *n* (%)	3 (20)
Female, *n* (%)	12 (80)
Ethnicity	
Hispanic, *n* (%)	15 (100)
Analysis eye	
Right, *n* (%)	8 (53.3)
Left, *n* (%)	7 (46.7)
Ocular signs, analysis eye	
Erythema, *n* (%)	
Severe	1 (6.7)
Moderate	3 (19.9)
Mild	10 (66.7)
None	1 (6.7)
Collarette grade, upper lid	
Mean (SEM)	3.07 (0.21)
Mite density (mites per lash)	
Mean (SEM)	2.28 (0.16)

## Data Availability

The data used to support the findings of this study are included within the article. Clarifications or additional data used to support the findings of this study may be requested from the corresponding author.

## References

[1] American Academy of Ophthalmology Cornea/External Disease Panel (2018). *Preferred Practice Pattern Guidelines. Blepharitis*.

[2] Zhao Y.-E., Wu L.-P., Hu L., Xu J.-R. (2012). Association of blepharitis with *Demodex*: a meta-analysis. *Ophthalmic Epidemiology*.

[3] Biernat M. M., Rusiecka-Ziółkowska J., Piątkowska E., Helemejko I., Biernat P., Gościniak G. (2018). Occurrence of *Demodex* species in patients with blepharitis and in healthy individuals: a 10-year observational study. *Japanese Journal of Ophthalmology*.

[4] Cheng A. M. S., Sheha H., Tseng S. C. G. (2015). Recent advances on ocular *Demodex* infestation. *Current Opinion in Ophthalmology*.

[5] Fromstein S. R., Harthan J. S., Patel J., Opitz D. L. (2018). *Demodex* blepharitis: clinical perspectives. *Clinical Optometry*.

[6] Luo X., Li J., Chen C., Tseng S., Liang L. (2017). Ocular demodicosis as a potential cause of ocular surface inflammation. *Cornea*.

[7] Post C. F., Juhlin E. (1963). *Demodex* folliculorum and blepharitis. *Archives of Dermatology*.

[8] Liu J., Sheha H., Tseng S. C. (2010). Pathogenic role of *Demodex* mites in blepharitis. *Current Opinion in Allergy and Clinical Immunology*.

[9] Zhong J., Tan Y., Li S. (2019). The prevalence of *Demodex* folliculorum and *Demodex* brevis in cylindrical dandruff patients. *Journal of Ophthalmology*.

[10] Gao Y.-Y., Di Pascuale M. A., Li W. (2005). High prevalence of *Demodex* in eyelashes with cylindrical dandruff. *Investigative Opthalmology & Visual Science*.

[11] Arrúa M., Samudio M., Fariña N. (2015). Comparative study of the efficacy of different treatment options in patients with chronic blepharitis. *Archivos de la Sociedad Espanola de Oftalmologia*.

[12] Murphy O., O’Dwyer V., Lloyd-McKernan A. (2018). The efficacy of tea tree face wash, 1, 2-Octanediol and microblepharoexfoliation in treating *Demodex* folliculorum blepharitis. *Contact Lens and Anterior Eye*.

[13] Gao Y.-Y. (2005). In vitro and in vivo killing of ocular *Demodex* by tea tree oil. *British Journal of Ophthalmology*.

[14] Karakurt Y., Zeytun E. (2018). Evaluation of the efficacy of tea tree oil on the density of *Demodex* mites (acari: demodicidae) and ocular symptoms in patients with demodectic blepharitis. *Journal of Parasitology*.

[15] Messaoud R., El Fekih L., Mahmoud A. (2019). Improvement in ocular symptoms and signs in patients with *Demodex* anterior blepharitis using a novel terpinen-4-ol (2.5%) and hyaluronic acid (0.2%) cleansing wipe. *Clinical Ophthalmology (Auckland, N.Z.)*.

[16] Savla K., Le J. T., Pucker A. D. (2020). Tea tree oil for *Demodex* blepharitis. *Cochrane Database of Systematic Reviews*.

[17] Hayes A. J., Leach D. N., Markham J. L., Markovic B. (1997). In vitro cytotoxicity of Australian tea tree oil using human cell lines. *Journal of Essential Oil Research*.

[18] Loughlin R., Gilmore B. F., McCarron P. A., Tunney M. M. (2008). Comparison of the cidal activity of tea tree oil and terpinen-4-ol against clinical bacterial skin isolates and human fibroblast cells. *Letters in Applied Microbiology*.

[19] Chen D., Wang J., Sullivan D. A., Kam W. R., Liu Y. (2020). Effects of terpinen-4-ol on meibomian gland epithelial cells in vitro. *Cornea*.

[20] Toutain C. E., Seewald W., Jung M. (2018). The intravenous and oral pharmacokinetics of lotilaner in dogs. *Parasites & Vectors*.

[21] https://www.fda.gov/animal-veterinary/animal-health-literacy/fact-sheet-pet-owners-and-veterinarians-about-potential-adverse-events-associated-isoxazoline-flea.

[22] European Medicines Agency https://www.ema.europa.eu/en/documents/product-information/credelio-epar-product-information_en.pdf.

[23] Ozoe Y., Asahi M., Ozoe F., Nakahira K., Mita T. (2010). The antiparasitic isoxazoline A1443 is a potent blocker of insect ligand-gated chloride channels. *Biochemical and Biophysical Research Communications*.

[24] Rufener L., Danelli V., Bertrand D., Sager H. (2017). The novel isoxazoline ectoparasiticide lotilaner (Credelio): a non-competitive antagonist specific to invertebrates *γ*-aminobutyric acid-gated chloride channels (GABACls). *Parasites & Vectors*.

[25] Vehige J. G., Baba S. N., Cruz V. S., Holdbrook M., Hom M. A new treatment strategy for *Demodex* infestation using topic antiparasitic isoxazoline drugs: results of ex vivo testing.

[26] Perks D. (2015). AHC 2224920: 13 Week oral (gavage) administration toxicity study in the rat followed by a 4-week treatment-free period.

[27] Koo H., Kim T. H., Kim K. W., Wee S. W., Chun Y. S., Kim J. C. (2012). Ocular surface discomfort and *Demodex*: effect of tea tree oil eyelid scrub in *Demodex* blepharitis. *Journal of Korean Medical Science*.

[28] Gao Y.-Y., Di Pascuale M. A., Elizondo A., Tseng S. C. G. (2007). Clinical treatment of ocular demodecosis by lid scrub with tea tree oil. *Cornea*.

[29] Qiu T. Y., Yeo S., Tong L. (2018). Satisfaction and convenience of using terpenoid-impregnated eyelid wipes and teaching method in people without blepharitis. *Clinical Ophthalmology*.

[30] Ngo W., Jones L., Bitton E. (2018). Short-term comfort responses associated with the use of eyelid cleansing products to manage *Demodex* folliculorum. *Eye and Contact Lens: Science and Clinical Practice*.

[31] Rutherford T., Nixon R., Tam M., Tate B. (2007). Allergy to tea tree oil: retrospective review of 41 cases with positive patch tests over 4.5 years. *Australasian Journal of Dermatology*.

[32] Cheng S.-n., Jiang F.-g., Chen H., Gao H., Huang Y.-k. (2019). Intense pulsed light therapy for patients with meibomian gland dysfunction and ocular *Demodex* infestation. *Current Medical Science*.

[33] Zhang X., Song N., Gong L. (2019). Therapeutic effect of intense pulsed light on ocular demodicosis. *Current Eye Research*.

[34] Kim J. H., Chun Y. S., Kim J. C. (2011). Clinical and immunological responses in ocular demodecosis. *Journal of Korean Medical Science*.

